# Antioxidant Characterization of Oak Extracts Combining Spectrophotometric Assays and Chemometrics

**DOI:** 10.1155/2013/134656

**Published:** 2013-12-25

**Authors:** Boris M. Popović, Dubravka Štajner, Ružica Ždero, Saša Orlović, Zoran Galić

**Affiliations:** ^1^Faculty of Agriculture, University of Novi Sad, Trg Dositeja Obradovića 8, 21000 Novi Sad, Serbia; ^2^Institute of Lowland Forestry and Environment, University of Novi Sad, Antona Čehova 13, 21000 Novi Sad, Serbia

## Abstract

Antioxidant characteristics of leaves, twigs, and acorns from two Serbian oak species *Quercus robur* L. and *Quercus petraea* L. from Vojvodina province (northern Serbia) were investigated. 80% ethanol (in water) extracts were used for antiradical power (ARP) determinations against DPPH^•^, ^•^NO, and O_2_^•−^ radicals, ferric reducing antioxidant power (FRAP), total phenol, tannin, flavonoid, and proanthocyanidin contents. Permanganate reducing antioxidant capacity (PRAC) was determined using water extracts. Beside, mentioned parameters, soluble proteins, lipid peroxidation (LP), pigments and proline contents were also determined. The data of different procedures were compared and analyzed by multivariate techniques (correlation matrix calculation and principal component analysis (PCA)). PCA found that investigated organs of two different oak tree species possess similar antioxidant characteristics. The superior antioxidant characteristics showed oak leaves over twigs and acorns and seem to be promising source of antioxidants with possible use in industry and pharmacy.

## 1. Introduction

Quercus trees, commonly known as oaks, belong to the family Fagaceae. They comprise 450 species worldwide [1]. European oak corresponds well with these requirements and is mainly represented by *Quercus robur* L. (pedunculate) and *Quercus petraea* (Matt.) Liebl. (sessile oak). Oak wood is valued for its mechanical properties and durability. It has been widely used since prehistoric times [2]. Pedunculate is the dominant tree species of natural forests in the area of flat Srem and also in whole region of Vojvodina, northern Serbia. Besides pedunculate, sessile oak is the most valuable oak in Serbia [3]. In the forestry fund of Serbia, there is the significant participation of sessile oak (7.28%) [4]. Oaks are the major source of hardwood lumber and also they are used for ornaments. The wood is durable and tough and also attractively grained. It is especially valued in shipbuilding and construction and for flooring, furniture, barrels, and veneer. The bark of some oaks has been used in medicine, in tanning, and for dyes. In mythology and religion, the oak was revered as a symbol of power [5]. In Serbia, oak is a sacred tree, used in Serbian Christmas traditions.

Acorns, the fruit of oak trees, have long been employed as a source of hog feed, tannin, oil, and especially food because of the high content of carbohydrates, amino acids, proteins, lipids, and various sterols [6, 7]. *Quercus* acorns were mainly used for making bread or as a substitute for coffee. Oak kernels were traditionally used in medicine, particularly roasted ones as astringents, antidiarrhoeals, and antidotes [8]. The acorns of *Q. robur* contain various biologically active compounds with antioxidant activity (tannins, gallic and ellagic acid, and different galloyl and hexahydroxydiphenoyl derivatives) [8, 9]. It was also known that the bark of different *Quercus* species contains polyphenolic constituents. Thus, the bark of *Q. petraea* contains both hydrolysable and condensed tannins, flavanols, and oligomeric proanthocyanidins [10]. From the bark of *Quercus robur* more than 20 compounds (catechins and oligomeric and polymeric proanthocyanidins) have been isolated [11].

Polyphenols are secondary metabolites of plants that are generally involved in defense against ultraviolet radiation and pathogens. In food, polyphenols contribute to the color, flavor, odor, bitterness, astringency, and oxidative stability. Recent biomedical investigations connected to the polyphenols and antioxidant activity of a number of herbals and foods show that polyphenols such are flavonoids, tannins, and proanthocyanidins offer protection against development of cancers, cardiovascular diseases, diabetes, osteoporosis and neurodegenerative diseases [12, 13].

Kim et al. [14] investigated phenolic profile in leaves of five different *Quercus* species and approved presence of many chemical constituents. This study demonstrated differences in phenolic compounds content in different parts of plants. *Quercus salicina *Blume, for example, possesses high levels of gentisic and chlorogenic acids as well as flavonoids naringin and rutin in the leaf. Brossa et al. [15] established that major constituents in holm oak (*Quercus ilex* L.) leaves are flavanols and flavonols. According to Kamalak et al. [16], oak leaves from some *Quercus *species (*Q. branti *and *Q. libari*) may have a high potential nutritive value for small ruminant animals in terms of rumen and whole tract digestion. It is established that content of phenolic compounds in *Quercus *species highly depends on the stage of maturity and expresses seasonal variation [15, 17].

Sanchez-Burgos et al. [1] established that aqueous extracts from leaves from different white *Quercus *species (*Quercus resinosa, Quercus laeta, Quercus grisea, *and* Quercus obtusata*) displayed high radical scavenging activity against DPPH and ^•^OH radicals as well as antimicrobial activities and antitopoisomerase activity only *Q. resinosa* leaves infusions. Andrenšek et al. [18] also pointed out that *Q. robur* cortex is a promising plant material as the source of antioxidative and antimicrobial activity.

Bearing in mind that antioxidant potential of two major Serbian oaks *Quercus robur* L. and *Quercus petraea *L. has not been studied well enough, especially their leaves and twigs, the aim of this work was to investigate the *in vitro* antioxidant and scavenging activities and also total phenol (TPC), tannin (TAC), flavonoid (FLC), Proanthocyanidin (PAC), and proline contents as well as lipid peroxidation (LP) in leaves, twigs, and acorns of these two Serbian oak tree species.

## 2. Materials and Methods

### 2.1. Plant Material and Extraction Procedure

This paper presents antioxidant characteristics of two Serbian oak species *Quercus robur* L. and *Quercus petraea *L. from Vojvodina province, in northern Serbia. During September 2011, twigs, leaves, and acorns were picked to make average samples (from 20 trees per one replicate). Three independent replicates were made for both species. All samples were dried in open air in the dark.

After that, 20 g of the dried sample was finely ground into a fine powder in a mill and extracted with 500 mL of water for 24 h at 25°C, followed by filtration. Prepared extract was used for lipid peroxidation, soluble protein, and PRAC determination. For all ARP determinations and FRAP the similar extraction tool was used, with 80% EtOH (in water) as an extractant. For TPC and TAC acidic ethanol (0.1 mol/dm^3^ HCl in EtOH) was used as an extractant. For determination of DPPH^•^, ^•^NO, and O_2_^•−^ ARP, 80% EtOH extracts were evaporated to dryness and the dry residues were redissolved again in 80% EtOH (in water) to obtain mass concentration 25 mg/mL (for DPPH^•^, ^•^NO and O_2_^•−^ ARP determination).

### 2.2. Lipid Peroxidation, Proline, Soluble Protein, and Pigment Contents

Lipid peroxidation (LP) was estimated based on thiobarbituric acid (TBA) reactivity. Samples were evaluated for malondialdehyde (MDA) production using a spectrophotometric assay. The extinction coefficient of 153,000 mol^−1^ cm^−1^ at 532 nm for the chromophore was used to calculate the colour intensity of the MDA-TBA complex in the supernatant [19].

Proline accumulation was determined by the method as described by Paquin and Lechasseur [20]. Proline was determined after extraction with sulphosalicylic acid, and reaction with ninhydrin. A standard curve of proline was used for calibration and was measured by its absorbance at 532 nm.

Pigments were extracted with acetone and determined spectrophotometrically using molar extinction coefficients according to von Wettstein [21]. Soluble protein content was determined by the method of Bradford [22].

### 2.3. Total Phenol, Tannin, Flavonoid, and Proanthocyanidin Contents

Total polyphenols were determined by Folin-Ciocalteu procedure [23]. The amount of total polyphenols was calculated as a catechin equivalent from the calibration curve of catechin standard solutions (covering the concentration range between 0.1 and 1.0 mg/mL) and expressed as mg catechi/100 g dry plant material.

Total tannin content was determined by Folin-Ciocalteu procedure as above, after removal of tannins by their adsorption on insoluble matrix (polyvinylpolypyrrolidone, PVPP). Calculated values were subtracted from total polyphenol contents and total tannin contents expressed as mg catechine/100 g dry plant material.

Total flavonoids were determined after extraction of plant material (1 g) with extracting solvent methanol-water-acetic acid (140 : 50 : 10, V/V), according to Markham [24]. The amount of flavonoids was calculated as a rutin equivalent from the calibration curve of rutin standard solutions and expressed as mg rutin/100 g of plant material.

Proanthocyanidins were determined by butanol-HCl assay [23]. Their contents were expressed as mg leucoanthocyanidin/100 g of dry plant material, assuming that the specific absorbance of leucoanthocyanidin was 460.

All measurements were done in triplicate.

### 2.4. FRAP

Total antioxidant capacity was estimated according to the ferric reducing antioxidant power (FRAP) assay [25]. FRAP reagent was prepared by mixing acetate buffer (300 mM pH 3.6), TPTZ (2,4,6-tripyridyl-s-triazine) reagent (10 mM in 40 mM HCl), and FeCl_3_·6H_2_0 (20 mM) in ratio 3 : 1 : 1. Sample (100 *μ*L) was mixed with 3 mL of working FRAP reagent and absorbance (593 nm) was measured at 4 minutes after vortexing. FRAP value was calculated using formula

(1) $$\begin{array}{c} \text{FRAP}\;\text{value}=\frac{\Delta A_{\text{sample}}\;\left(\text{0–4 min}\right)}{\Delta A_{\text{standard}}\;\text{(0–4 min)}}. \end{array}$$

100 *μ*M Fe^2+^ was used as a standard; 1 FRAP unit = 100 *μ*M Fe^2+^.

Total antioxidant capacity was expressed in FRAP units.

### 2.5. Permanganate Reducing Antioxidant Capacity

The method is based on the redox reactions between the antioxidant sample and the potassium permanganate in sulfuric acid media, leading to sample discoloration until no colour is observed [26]. Variable amounts of samples (*V*-mL), depending on the intensity of the antioxidant activity, were introduced in a 30 mL quartz vat containing an oxidative mixture of 1.5 mL potassium permanganate 0.01 M; 3.5 mL sulfuric acid 2 M, and (20-*V*) mL distilled water. That moment was considered the zero time. The spectrophotometer signal was then registered at 535 nm until constant value. Subsequent decrease of potassium permanganate concentration was determined based on a previously prepared calibration curve. A calibration curve was determined by preparing a series of six solutions with different concentrations of potassium permanganate and registering the absorbance for each of them. In order to quantitatively compare the antioxidant activities, we proposed the following formula:

(2) $$\begin{array}{c} A_{50}=\frac{t_{\left(\text{standard}\right)}}{t_{\left(\text{plant}\;\text{sample}\right)}}\cdot \frac{c_{\left(\text{standard}\right)}}{m_{\left(\text{plant}\right)}}\cdot \frac{V_{\left(\text{standard}\right)}}{V_{\left(\text{plant}\;\text{sample}\right)}}\cdot V_{\left(\text{extract}\right)}, \end{array}$$

where *A*_50_ is antioxidant activity expressed, reflected in the time until the sample induces a decrease of the oxidizing agent [potassium permanganate] concentration up to one half, compared against a standard [ascorbic acid] (mmol equivalent standard/g plant), *t*_(plant  sample)_ is the time until the sample induces a decrease of the permanganate concentration up to one half (min), *t*_(standard)_ is the time until the standard (ascorbic acid) induces a decrease of the permanganate concentration up to one half (min), *c*_(standard)_ is standard (ascorbic acid) concentration (mmol/mL) [0.01 mmol/mL], *m*_(plant)_ is weight (g) of the plant sample submitted to extraction [4 g], *V*_(plant  sample)_ is volume of the plant extract submitted to the analysis [0.2 mL], *V*_(standard)_ is volume of the standard submitted to the analysis [1 mL], and *V*_(extract)_ is volume (mL) of the obtained extract [40 mL].

### 2.6. Radical Scavenging Determinations

DPPH^•^-RSC assay was based on measurement of the loss of DPPH (2,2-diphenyl-1-picrylhydrazyl) color after reaction with test compounds [27]. The DPPH^•^ radical is one of the few stable organic nitrogen radicals, which bears a deep purple color. This assay is based on the measurement of the reducing ability of antioxidants toward DPPH^•^. The ability can be evaluated by measuring the decrease of its absorbance. The widely used decoloration assay was first reported by Brand-Williams et al. [28]. Appropriate volume of each extract was mixed with 90 *μ*M DPPH^•^ in methanol making up final volume of 3.0 mL. The mixtures were shaken vigorously and were stored in dark for 30 min at room temperature. The decrease of absorbance of the reaction mixtures regarding the control was monitored spectrophotometrically at 515 nm.

^•^NO-RSC was evaluated by measuring the accumulation of nitrite (formed by the reaction of NO with oxygen), according to the Griess reaction [29]. NO was generated by sodium nitroprusside in buffered aqueous solution. Appropriate volume of each extract was mixed with fresh prepared solution of sodium nitroprusside (0.5 mL, 0.01 M in NaH_2_PO_4_-Na_2_HPO_4_ buffer, 0.067 M, pH 7.4) and NaH_2_PO_4_-Na_2_HPO_4_ buffer (0.067 M, pH 7.4) making final volume of 1.0 mL. These mixtures were illuminated at 3000 lx and 25°C for 10 min. After illumination, each reaction mixture (1 mL) was mixed with Griess reagent (1 mL, 0.1% N-(1-naphtyl)-ethylenediamine dihydrochloride (NEDA) in distilled water and 1% sulfanilamide in 5% H_3_PO_4_). Reduction of nitrite by the extracts was determined spectrophotometrically at 546 nm, by measuring the decrease of absorbance of the reaction mixtures regarding the control (containing the same chemicals, except for the sample).

O_2_^•−^-RSC assay was based on the capacity of crude extracts to inhibit the photochemical reduction of nitro blue tetrazolium (NBT) in the riboflavin-light-NBT system [30]. Each 3 mL of reaction mixture contained sodium phosphate buffer (50 mM, pH 7.8), methionine (13 mM), riboflavin (2 *μ*M), EDTA (100 *μ*M), NBT (75 *μ*M), and extract solution. Reaction systems were illuminated at 3000 lx and 25°C for 10 min. The increase in absorbance at 560 nm was monitored. The scavenging capacity was expressed as reduction percentage of NBT absorbance induced by sample.

For each sample three replicates were carried out. RSC was calculated by the following equation:

(3) $$\begin{array}{c} \text{RSC}=\left(\frac{A_{0}-A_{1}}{A_{0}}\right)\cdot 100, \end{array}$$

where *A*_0_ is control and *A*_1_ is a sample solution absorbance. The concentration (in the final reaction media in each method) that causes a decrease in the initial absorbance (control) by 50% is defined as IC_50_. The IC_50_ values for all RSC determinations were determined by polynomial fitting of the inhibition values (RSC) using software ORIGIN 9.1. The antioxidant capacity of the extracts was expressed as antiradical power (ARP) and it was defined as

(4) $$\begin{array}{c} \text{ARP}=\left(\frac{1}{{\text{IC}}_{50}}\right)\cdot 100. \end{array}$$

### 2.7. Statistical and PCA Analysis

Statistical comparisons between samples were performed with Duncan *t*-test for independent observations. Differences were considered significant at *P* < 0.05. The antioxidant test results were investigated with multivariate analysis. The correlation matrix was calculated, giving the correlation coefficients between each pair of variables, that is, the analytical parameters tested. Each term of the matrix is a number ranging from −1 to +1: the + or − sign indicates a positive or negative interdependence between variables (direction), and the absolute value indicates the strength of the interdependence. Correlations between different parameters were considered significant at *r* > 0.95 (*P* < 0.05). Autoscaling transformation of data for phenolic parameters (TPC, TAC, FLC, and PAC) was done using STATISTICA 9.1 and presented by graphic (Figure 2).

## 3. Results and Discussion

### 3.1. Soluble Proteins, Proline, Pigment and MDA Contents

Soluble protein content ranged from 52.45 (*Q. robur* twigs) to 427.0 mg/g (*Q. robur* leaves); Proline content ranged from 0.041 (*Q. petraea* acorns) to 0.083 *μ*g/g (*Q. robur* twigs); Chla content ranged from 0.016 (*Q. robur* acorns) to 0.229 mg/g (*Q. petraea* leaves); Chlb content ranged from 0.017 (*Q. robur* acorns) to 0.164 mg/g (*Q. petraea* leaves); Carotenoid content ranged from 0.009 (*Q. robur* acorns) to 0.175 mg/g (*Q. robur* leaves); MDA content ranged from 2.023 (*Q. petraea* acorns) to 67.18 nmol/mg protein (*Q. robur* twigs), (Tables 1 and 2). Significant positive correlations were found between Chla and Chlb (*r* = 0.9957), and also between carotenoids and both chlorophylls (*r* = 0.84). Positive correlation was found between proline content and MDA content (*r* = 0.4459).

Lipids are susceptible to oxidation and lipid peroxidation products, such as MDA quantity, are potential biomarkers for oxidative stress status *in vivo*. Proteins are also the direct target for Reactive Oxygen Species (ROS) because of their high concentrations. Their oxidation may result in deamination, decarboxylation, peptide backbone cleavage, cross-linking, and many other chemical modifications leading eventually to inactivation of enzyme activity and accumulation within cells and extracellular environment [31]. Furthermore, antioxidant capacity and the ratio between reduced forms to oxidized forms of molecules may be also used as biomarkers of oxidative stress [32]. According to our results, the highest accumulation of MDA was observed in twigs of both *Quercus* species, where the soluble protein content was lowest due to increased level of oxidative stress.

Carotenoids, along with phenolics, are responsible for bright colours of plants and are also powerful antioxidants. Carotenoids can protect membranes against damage by free radicals and retard the ageing processes [33]. The highest content of pigments, carotenoids, and chlorophylls was found in the leaves of both, especially *Q. petraea*. Proline is an amino acid that acts as an antioxidant—it reduces free radicals in plant cells [34, 35]. Its production is a self-defense mechanism. Plant's proline levels are an indicator of both the environment stress and the plant's response. Proline does not interfere with normal biochemical reactions but allows the plants to survive under stress [36]. The accumulation of proline in plant tissues is also a clear marker for environmental stress, particularly in plants under drought conditions and may also be part of the stress signal influencing adaptive responses [37]. Positive correlation between free proline content and LP intensity confirms both antioxidant and defense natures of this amino acid.

### 3.2. Total Phenol, Tannin, Flavonoid, and Proanthocyanidin Contents

Total phenol content ranged from 7.44 (*Q. petraea* twigs) to 35.52 mg catechin/g (*Q. petraea* leaves); Tannin content ranged from 4.667 (*Q. petraea* twigs) to 26.24 mg catechin/g (*Q. petraea* leaves); Flavonoid content ranged from 19.12 (*Q. petraea* twigs) to 306.1 mg rutin/100 g (*Q. petraea* leaves); Proanthocyanidine content ranged from 79.04 (*Q. petraea* twigs) to 1102 mg rutin/100 g (*Q. robur* leaves) (Table 1). Significant positive correlations were observed between all mentioned parameters. The highest positive correlation was observed between TPC and TAC (*r* = 0.9955). All phenolic parameters were significantly positively correlated with carotenoid content. Total phenol, tannin, and flavonoid contents were also significantly positively correlated with both chlorophylls (a and b). Phenolic parameters were analyzed by a multivariate approach and results are showed by line plot of multiple variables (Figure 3). All phenolic parameters are negatively but not significantly correlated with LP parameter.

Our results are in accordance with that obtained by Rakić et al. [38] who indicated that oak acorns from *Q. robur* are material rich in polyphenols and tannins. We have found similar results for *Q. petraea* acorns. Kamalak et al. [16] evaluated nutritive values of browse leaves from five oak species, namely, *Quercus branti*, *Quercus coccifera*, *Quercus cerris*, *Quercus libari*, and *Quercus infectoria *based on their chemical composition. It was established that tannin content ranged from 14.9 to 47.9 mg/g matter which is in accordance with our results for *Q. robur* and* Q. petraea*. *Q. petraea* and* Q. robur* are also rich source of flavonoids and proanthocyanidins which are found in all plant organs, especially in leaves. Flavonoid content found in leaves of *Q. petraea *and* Q. robur* is in range of that found in *Quercus salicina* Blume [39]. According to Salminen et al. (2004) hydrolysable tannins were the dominant phenolic group in leaves of all ages of *Q. robur* which is in well agreement with our results. However, hydrolysable tannins and flavonoid glycosides showed highly variable seasonal patterns. Young oak leaves were much richer in hydrolysable tannins and flavonoid glycosides than old leaves, and *vice versa* for proanthocyanidins [40]. Although in smaller quantities, twigs also contained all classes of polyphenols (Table 1). The obtained results have provided further grounds for establishing *Q. robur *and* Q. petraea* leaves, acorns, and twigs as a source for functional food preparation.

### 3.3. FRAP and PRAC Methods

FRAP values ranged from 141.54 (*Q. robur* twigs) to 1252.3 FRAP units (*Q. petraea* leaves); PRAC values ranged from 0.010 (*Q. petraea* twigs) to 0.543 mmol ascorbate eq./g (*Q. petraea* acorns) (Table 2). FRAP was significantly positively correlated with phenolic parameters (TPC, TAC, and FLC) and with pigment (Chla, Chlb, and Car) contents. The highest positive correlation was observed between FRAP and TAC (*r* = 0.9587). PRAC value was positively correlated with O_2_^•−^-ARP (*r* = 0.6196) and negatively correlated with LP (*r* = −0.8113).

Benzie and Strain [25] introduced FRAP as a simple and automated test measuring the ferric reducing ability of plasma and a novel method for assessing “antioxidant power.” Ferric to ferrous ion reduction at low pH causes a colored ferrous-tripyridyltriazine complex to form. According to Maqsood and Benjakul [41] tannic acid showed higher FRAP value in comparison with other investigated phenolics including catechin which is in agreement with our observation that the highest positive correlation was found between FRAP and TAC parameter. Same reaction mechanism based on electron transfer explains high positive correlations between FRAP and phenolic parameters (TPC, TAC, and FLC) [42]. According to FRAP method, antioxidant capacity of oak samples, especially leaves, was relatively high comparing with other plants [43–45]. FRAP value was more or less positively correlated with all investigated parameters excluding LP, where negative correlation was found (−0.5448).

PRAC method was firstly introduced by Cacig and Szabo [26] as a simple spectrophotometric method for evaluation of antioxidant capacity and later compared with other total antioxidant capacity methods [46]. Unlike the oak leaves where the highest FRAP was found, oak acorns showed the highest PRAC. High positive correlations with O_2_^•−^-ARP indicate that the same structures are probably responsible for superoxide scavenging and for reduction of permanganate in acidic media. The highest negative correlations of both parameters with LP also indicate that scavengers of superoxide and substances with high reducing potential against permanganate are mostly responsible for suppression of LP.

### 3.4. Antiradical Power Determinations

DPPH-ARP ranged from 4.039 (*Q. petraea* twigs) to 9.066 (*Q. robur* acorns); NO-ARP ranged from 0.188 (*Q. robur* acorns) to 0.531 (*Q. robur* leaves); O_2_^•−^-ARP ranged from 2.174 (*Q. robur* twigs) to 5.359 (*Q. robur* acorns) (Table 2). DPPH-ARP and NO-ARP were positively correlated with all phenolic parameters (TPC, TAC, FLC, and PAC), pigments (Chla, Chlb, and Car), and FRAP. In contrast to them, O_2_^•−^-ARP showed rather less positive correlations with phenolic parameters, pigments, and FRAP but relatively high positive correlation with PRAC (0.6196). Among investigated ARP parameters, only O_2_^•−^-ARP showed significant negative correlation with LP (*r* = −0.8360).

All three ARP power determinations against DPPH, O_2_^•−^, and NO radicals generally proceed also *via* hydrogen atom transfer or electron transfer mechanism depending on present antioxidant structure, pH, dielectric constant of the solvent, and so forth [46]. DPPH and O_2_^•−^-ARP were correlated with each other (*r* = 0.6776), while correlations with NO-ARP were much lower. Significant positive correlation of NO-ARP and carotenoids (*r* = 0.8525) indicated significance of carotenoid antioxidants for NO scavenging. Sindhu et al. [47] also established that carotenoids lutein and zeaxanthin showed stronger antiradical potential against NO than DPPH and O_2_^•−^ radicals. The highest DPPH- and O_2_^•−^-ARP showed acorns and the highest NO-ARP showed leaves *Q. robur*. Rivas-Arreola et al. [48] also investigated antioxidant activity of oak (*Q. sideroxyla*, *Q. Eduardii*, and *Q. resinosa*) leaves infusions against free radicals and obtained similar results for radical scavenger capacities. Oak twigs expressed lower DPPH, O_2_^•−^, and NO ARP in comparison with leaves and acorns but also relatively high antiradical potential.

### 3.5. PCA Analysis

**Figure 1:**
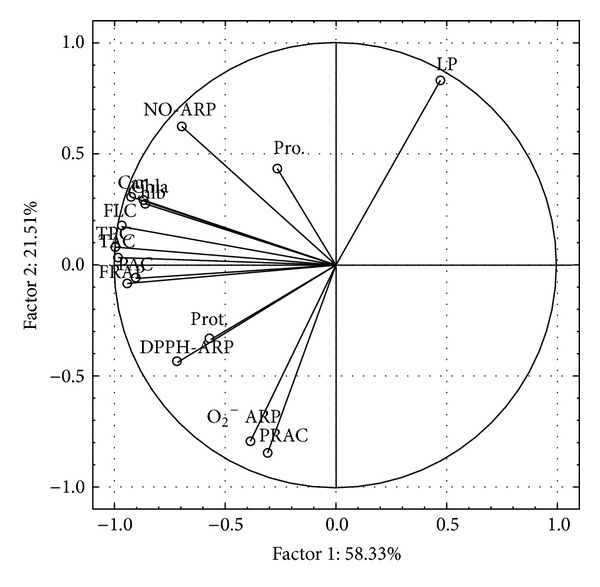
Graph of loading plot of antioxidant markers for Serbian oak species *Quercus robur* L. and *Quercus petraea *L. Prot.: proteins; ARP: antiradical power; PRAC: permanganate reducing antioxidant capacity; FRAP: ferric reducing antioxidant power; LP: lipid peroxidation; TPC: total phenolic content; TAC: tannin content; FLC: flavonoid content; PAC: Proanthocyanidin content. Chla and Chlb: chlorophyll a and b contents; car: Carotenoid content; Pro: proline content. Parameters with close interdependence and correlation are close to each other and vice versa.

**Figure 2:**
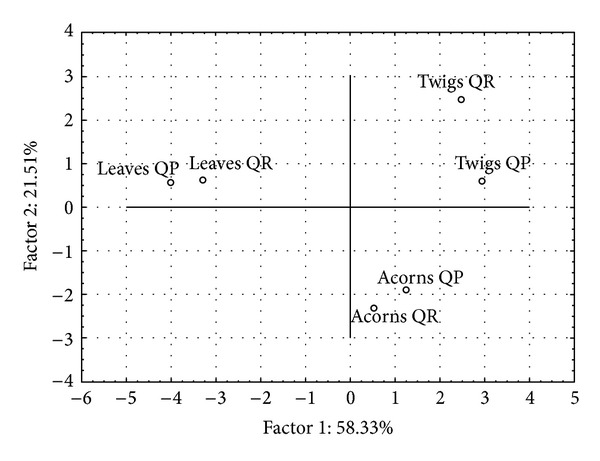
Graph of scores plot for Serbian oak species *Quercus robur* L. (QR) and *Quercus petraea *L. (QP). *Quercus robur* L. (QR) and* Quercus* samples that are close to each other possess similar antioxidant statuses.

The original data set was renormalized by an autoscaling transformation (data not shown) and different parameters were analyzed by a multivariate approach. The loadings plot is presented by Figure 1 and the scores plot by Figure 2. The scree plot (data not shown) indicates that the first two principal components account for 79.84% of the total variance (PC1 = 58.33 and PC2 = 21.51).

As reported in the loadings plot (Figure 1), antiradical power parameters (DPPH^•^, ^•^NO, and O_2_^•−^-ARP), FRAP, polyphenol (TPC, TAC, FLC, and PAC), protein, and pigment contents are positioned closely due to the significant positive correlations among them. PRAC and O_2_^•−^ ARP are partially isolated and located opposite to LP as a parameter of oxidative stress. Positive correlations were also found among DPPH-ARP and proteins and also between ^•^NO-ARP and proline content.

**Figure 3:**
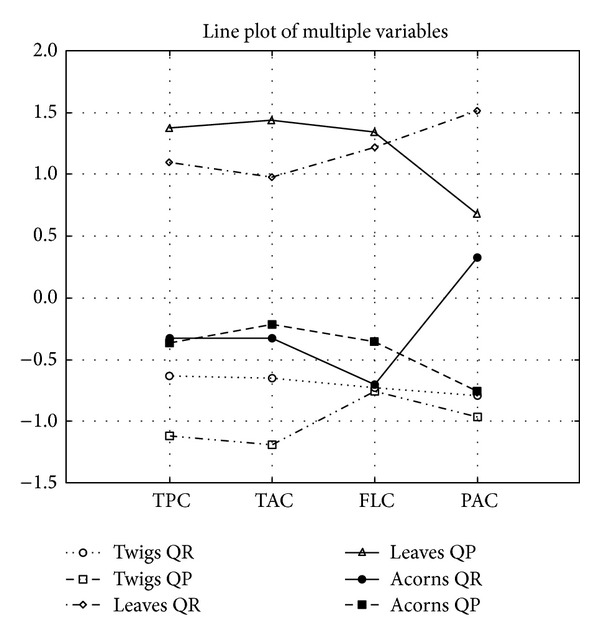
Line plot of multiple variables after autoscaling transformation of phenolic content parameters (TPC, TAC, FLC, and PAC). *Quercus robur* L. (QR) and *Quercus petraea *L. (QP). TPC: Total phenolic content; TAC: Tannin content; FLC: Flavonoid content; PAC: Proanthocyanidin content.

PCA found three different clusters of oak samples based on antioxidant characteristics: leaves, acorns, and twigs from investigated species are grouped (Figure 2). Leaves differ from twigs and acorns predominantly by Factor 1 (where the major contributors are polyphenols and FRAP). And the difference between twigs and acorns is based on Factor 2 (where the major contributors are LP, PRAC, and O_2_^•−^-ARP). Opposite direction of LP on one side and PRAC O_2_^•−^-ARP on another side indicates that the major contributors against lipid peroxidation are components which are antioxidants which can easily reduce permanganate and also scavenge O_2_^•−^-radicals. Very close interdependence was observed between leaves and twigs from both species, but twigs from two *Quercus* species showed slightly higher differences. Line plot of multiple variables after autoscaling transformation of polyphenolic content parameters (TPC, TAC, FLC, and PAC) was shown in Figure 3. It is obvious that polyphenolic parameters for leaves are separated and are greater than the same parameters for acorns and twigs.

## 4. Conclusion

This investigation pointed out antioxidant potential of both Serbian oak species (*Q. robur* and *Q. petraea*). Leaves from both oak species possessed high contents of total phenols, tannins, flavonoids, proanthocyanidins, and pigment contents. Antiradical power parameters were also very high for oak leaves and LP intensity was relatively low. Ferric reducing antioxidant capacity was the highest in oak leaf extracts, especially for *Q. petraea*. Among investigated leaf extracts which are significant source of phenolic compounds, oak acorns showed also high antioxidant potential and the lowest LP intensity. Antioxidant capacity values including DPPH^•^, ^•^NO and O_2_^•−^-ARP, and FRAP showed high positive correlations among themselves and also with polyphenol parameters (TPC, TAC, FLC, and PAC), protein, and pigment contents. Permanganate PRAC and O_2_^•−^-ARP were selected as the best antioxidant markers for oak trees because of the highest negative correlations with LP intensity. Considering high antioxidant potential of investigated organs of Serbian oak species (*Q. robur* and *Q. petraea*), besides acorns, oak leaves, and even twigs, could be recommended as source of natural antioxidants and promising source of pharmaceuticals with possible use in industry and pharmacy.

## Figures and Tables

**Table 1:** Total phenol, tannin, flavonoid, proantocyanidine, chlorophyll a and b, carotenoid, and proline contents in oak twigs, leaves, and acorns of two Serbian oak species *Quercus robur* L. and *Quercus petraea *L.

Plant organ	Locality	TPC (mg catechin/g)	TAC (mg catechin/g)	FLC (mg rutin/100 g)	PAC (mg leukocyani-dine/100 g)	Chla (mg/g)	Chlb (mg/g)	Car (mg/g)	Pro. (*μ*g/g)
Twigs	*Q. robur *	12.86^a^	9.094^a^	22.06^a^	148.9^a^	0.033^a^	0.027^a^	0.029^a^	0.083^a^
	*Q. petraea *	7.44^b^	4.667^b^	19.12^a^	79.04^b^	0.035^a^	0.042^b^	0.018^b^	0.053^b^
Leaves	*Q. robur *	32.37^c^	22.47^c^	290.2^b^	1102^c^	0.115^b^	0.091^c^	0.175^c^	0.067^c^
	*Q. petraea *	35.52^d^	26.24^d^	306.1^c^	757.6^d^	0.229^c^	0.164^d^	0.147^d^	0.075^d^
Acorns	*Q. robur *	16.25^e^	11.76^e^	26.17^a^	612.4^e^	0.016^d^	0.017^e^	0.009^e^	0.077^ad^
	*Q. petraea *	15.90^e^	12.65^e^	73.81^d^	163.6^a^	0.026^a^	0.032^a^	0.023^ab^	0.041^e^

*Values with the same letter, in each colon, are not significantly different according to Duncan test (*P* < 0.05).

**TPC: total phenolic content; TAC: tannin content; FLC: flavonoid content; PAC: proanthocyanidin content; Chla and Chlb: chlorophyll a and b contents; Car: carotenoid content; Pro: proline content.

**Table 2:** Protein content, DPPH, NO, and O_2_^•−^-antiradical powers, permanganate reducing antioxidant capacity, ferric reducing antioxidant power, and lipid peroxidation in oak twigs, leaves, and acorns of two Serbian oak species *Quercus robur* L. and *Quercus petraea *L.

Plant organ	Locality	Prot. (mg/g)	DPPH-ARP ((1/IC_50_)·100)	NO-ARP ((1/IC_50_)·100)	O_2_^•−^-ARP ((1/IC_50_)·100)	PRAC (A_50_)	FRAP (FRAP units)	LP (nmol/mg prot.)
Twigs	*Q. robur *	52.45^a^	5.874^a^	0.358^a^	2.174^a^	0.016^a^	141.54^a^	67.18^a^
	*Q. petraea *	103.9^b^	4.039^b^	0.241^b^	3.571^b^	0.010^a^	178.5^a^	28.08^b^
Leaves	*Q. robur *	427.0^c^	7.628^c^	0.531^c^	3.448^b^	0.304^b^	873.8^b^	11.01^c^
	*Q. petraea *	159.6^d^	8.779^d^	0.400^d^	4.785^c^	0.235^c^	1252.3^c^	11.96^c^
Acorns	*Q. robur *	352.1^e^	9.066^d^	0.188^e^	5.359^d^	0.411^d^	370.0^d^	3.282^d^
	*Q. petraea *	95.60^b^	6.734^e^	0.202^e^	4.098^e^	0.543^e^	614.6^e^	2.023^d^

*Values with the same letter, in each colon, are not significantly different according to Duncan test (*P* < 0.05).

**Prot.: proteins; ARP: antiradical power; ARP = ((1/IC_50_)·100); IC_50_: the concentration of an sample at which 50% inhibition of free radical activity is observed; PRAC: permanganate reducing antioxidant capacity; A_50_: antioxidant activity reflected in time until the sample induces a decrease of the oxidizing agent (potassium permanganate) up to one half, compared against a standard (ascorbic acid); A_50_ = mmol ascorbate eq./g; FRAP: ferric reducing antioxidant power; 1 FRAP unit = 100 *μ*mol/dm^3^ Fe^2+^; LP: lipid peroxidation.

## Abbreviations

PCA: Principal component analysis
RSC: Radical scavenging capacity
ARP: Antiradical power
PRAC: Permanganate reducing antioxidant capacity
FRAP: Ferric reducing antioxidant power
LP: Lipid peroxidation
TPC: Total phenolic content
TAC: Tannin content
FLC: Flavonoid content
PAC: Proanthocyanidin content
Chla and Chlb: Chlorophyll a and b contents
Car: Carotenoid content
Pro: Proline content.
